# Impaired vibrotactile sense in children and adolescents with type 1 diabetes – Signs of peripheral neuropathy

**DOI:** 10.1371/journal.pone.0196243

**Published:** 2018-04-19

**Authors:** Erik Ising, Lars B. Dahlin, Helena Elding Larsson

**Affiliations:** 1 Department of Clinical Sciences—Pediatric Endocrinology, Lund University, Malmö, Sweden; 2 Department of Translational Medicine—Hand Surgery, Lund University, Malmö, Sweden; 3 Department of Hand Surgery, Skåne University Hospital, Malmö, Sweden; 4 Department of Pediatrics, Skåne University Hospital, Malmö, Sweden; Weill Cornell Medicine-Qatar, QATAR

## Abstract

**Objective:**

To investigate whether multi-frequency vibrometry can identify individuals with elevated vibration perception thresholds (VPTs), reflecting impaired vibrotactile sense, among children and adolescents with type 1 diabetes.

**Methods:**

In 72 pediatric patients with type 1 diabetes, VPTs were evaluated for seven frequencies on two sites of the hand, and five frequencies on two sites of the foot. Z-scores, based on previously collected reference data, were calculated. Perception to light touch was investigated using monofilaments. Subjects’ characteristics were analyzed in comparison to normal and impaired vibrotactile sense.

**Results:**

Subjects’ median age, disease duration and age at disease onset were 12.8, 5.3 and 6.9 years, respectively. A total of 13 out of 72 (18%) subjects had impaired vibrotactile sense on at least one foot site. Impaired vibrotactile sense was more common among subjects treated with multiple daily insulin injections (MDI) compared to subjects treated with continuous subcutaneous insulin infusion (CSII) (p = 0.013). Age at disease onset was higher among subjects with impaired vibrotactile sense (p = 0.046). No significant correlations were found with gender, HbA1c or duration of diabetes.

**Conclusions:**

Impaired vibrotactile sense, mirroring diabetic peripheral neuropathy, was found in 1/5 of the children and adolescents in the study, and was more common in patients treated with MDI than in subjects treated with CSII.

## Introduction

Peripheral neuropathy is a well-known complication to diabetes type 1 and 2 (T1D; T2D), that not only affects the patients’ physical health and ability to be physically active, but also influences their quality of life [1–3]. Furthermore, diabetic peripheral neuropathy (DPN) is the most important factor in the development of diabetic foot ulcers–a major health and economical issue worldwide [4–6]. A recent study estimated the cost of diabetic foot care in the UK in 2010–11 to £580 millions [7]. Although we do not have any cure for DPN today, it is important to find the subjects with DPN, in order to increase awareness that these patients are likely to develop diabetic foot ulcers in the future.

Children and adolescents have also been shown to, at least in cases of unsatisfying metabolic control, present with signs of DPN [8, 9]. Although these signs are visible, most of these children and adolescents are asymptomatic and the DPN is thus in a subclinical stage. When using electrophysiology, i.e. nerve conduction study, on children and adolescents, abnormalities have been detected in 28–58% of the cases [10]. The nerve conduction studies are the gold standard method for the diagnosis of DPN. However, they might be invasive, time consuming and not always easy to access. Thus, there is a need of accessible and sensitive screening methods for the early diagnosis of DPN [11, 12]. Measuring perception to vibrotactile stimulus with tuning forks or a biothesiometer and perception to light touch (LTP) with multiple monofilaments have been suggested as suitable methods [13]. However, studies testing these methods have given inconclusive results with widely varying sensitivity and specificity in comparison to nerve conduction studies [9, 10]. An article by Louraki et al. reported that screening for DPN using a biothesiometer resulted in a prevalence of abnormal vibrotactile sense varying between 6.2 and 62.5% between different studies [9]. The great difference in prevalence is thought to depend on different cut-off points, as well as, different definitions of diabetic neuropathy. An important issue with using a biothesiometer in screening for DPN is that the reproducibility is limited in comparison to nerve conduction studies; Louraki et al. report that the reproducibility was lowest among the subjects with the longest disease duration, the poorest metabolic control, as well as the presence of obesity [14]. Today, consensus is lacking on when to start, and how to perform, screening for DPN in children and adolescents [15].

The vibrotactile sense depends on the function of Meissner´s and Pacini´s corpuscles in the skin, reflecting large nerve fiber function. Using the vibrotactile sense to look for alterations mirroring impairments in the function of large nerve fibers is well established, and furthermore, the foundation of screening methods, such as tuning forks and biothesiometers [16]. Meissner´s corpuscles are most sensitive to frequencies around 30 Hz [17] and Pacini´s react mainly to vibrations around 150–250 Hz [18, 19]. With this in mind, a method stimulating both of these vibrotactile receptors would, at least theoretically, provide a more truthful picture of the status of vibrotactile sense in the glabrous skin. A previous study using the Vibrosense meter for the examination of vibration perception thresholds (VPTs) in the hands of adults with T1D in comparison with age and gender matched controls has shown that patients, 20 years after diagnosis, had higher VPTs in the hands, mainly at low frequencies, than controls [20]. The Vibrosense meter has several advantages compared to tuning forks and a biothesiometer, for example being user dependent instead of examiner dependent. The Vibrosense meter runs an examination depending on the response of the subject being examined, whereas a biothesiometer is dependent on the one holding the instrument. In order to use a biothesiometer or a tuning fork, the examiner needs to hold the device with a constant pressure to the skin. The Vibrosense meter on the other hand, gives an instant report, by turning on and off different led lights, when the subjects pressure on the vibrating probe is too hard or too soft [21].

There are no previous studies using the Vibrosense meter in children and adolescents with type 1 diabetes. However, there is a study on healthy children and adolescents representing the normal material of this study [21]. Furthermore, studies using the Vibrosense meter in adults have shown higher VPTs in adults with T1D and T2D compared to controls [22].

Our aim was to evaluate VPTs, obtained with a VibroSense Meter, and LTP, using Semmes-Weinstein’s monofilaments, in children and adolescents with T1D, in order to investigate if subjects with impaired sense, reflecting underlying sensory DPN, can be identified in relation to previously collected normative data [21]. We also aimed to investigate epidemiologic and clinical factors associated with impaired vibrotactile sense.

## Subjects and methods

### Subjects

Children and adolescents with T1D at the pediatric outpatient clinics at Skåne University Hospital and the Hospital of Helsingborg were, between April 2015 and June 2016, when visiting their pediatrician for regular follow up of their diabetes, asked for participation in this study. The regular follow up includes assessment of HbA1c values, measuring height and weight, and targeted physical examinations based on symptoms. Inclusion criteria: all patient interested in participating and diagnosed with T1D. Exclusion criteria: younger than eight or older than 18 years of age, subjects with non-analyzable curves, as well as subjects with other diseases than T1D, coeliac disease or autoimmune thyroiditis than can also give symptoms of impaired vibrotactile sense.

Eighty-two children and adolescents (boys = 43) accepted the invitation. Four subjects (boys = 2) did not meet the age criteria and five subjects (boys = 2) were excluded due to non-analyzable curves. One patient was excluded due to concomitant juvenile arthritis, treated with methotrexate. Eight patients (11%) suffered from concomitant celiac disease and six patients were having thyroid autoantibodies (8%), but only three of these (4%) where treated with levothyroxine. Furthermore, one of the subjects participated in the Etanercept Diamyd Combination Regimen study (ClinicalTrials.gov Identifier: NCT02464033). None of the included patients suffered from chronical diseases other than T1D, celiac disease or autoimmune thyroiditis. No subjects were excluded from participation or statistical analyses if they participated in other studies, or if they suffered from concomitant celiac disease or autoimmune thyroiditis.

The local ethics committee at Lund University approved the study (386/2007). Written informed consents were obtained from the legal guardian(s) of the children and adolescents participating in the study, as well as from the participants themselves. The research is conducted in accordance with the Declaration of Helsinki.

### Methods

All examinations were carried out by two research nurses, as described below and previously reported [21]. In half of the patients, VPT and LTP measurements were performed initially in the hand, followed by the foot and in the other half, measurements were performed in the opposite order, starting with the foot, with the intention to adjust for possible lack of compliance at the end of each examination.

#### Vibration perception thresholds

In short, VPTs are obtained by letting the subject push a button when vibrations, from a vibrating probe, are perceived on the hand or foot, and release the button when the vibration is no longer noticed. This results in curves with several endpoints, reflecting the button being pushed and released, as well as numerical values of the vibrotactile thresholds, for all frequencies and sites tested.

Examinations were performed in a secluded examination room, and the patients wore hearing protectors in order to maintain a calm and quiet environment [21]. VPTs were obtained from the finger pulps of index and little fingers in the right hand, reflecting the median and ulnar nerves respectively, at seven frequencies (8, 16, 32, 64, 125, 250 and 500 Hz), using a standard VibroSense Meter device (VibroSense Dynamics AB, Malmö, Sweden) [21]. On the foot, the measurements were done from the sole at the first and fifth metatarsal heads (MTH 1; MTH 5) of the right foot, reflecting the function of the medial and lateral branches of the tibial nerve, at five frequencies (8, 16, 32, 64 and 125 Hz) using a modified VibroSense Meter (VibroSense Dynamics AB, Malmö, Sweden) adopted for measurement on the feet [21].

**Fig 1:**
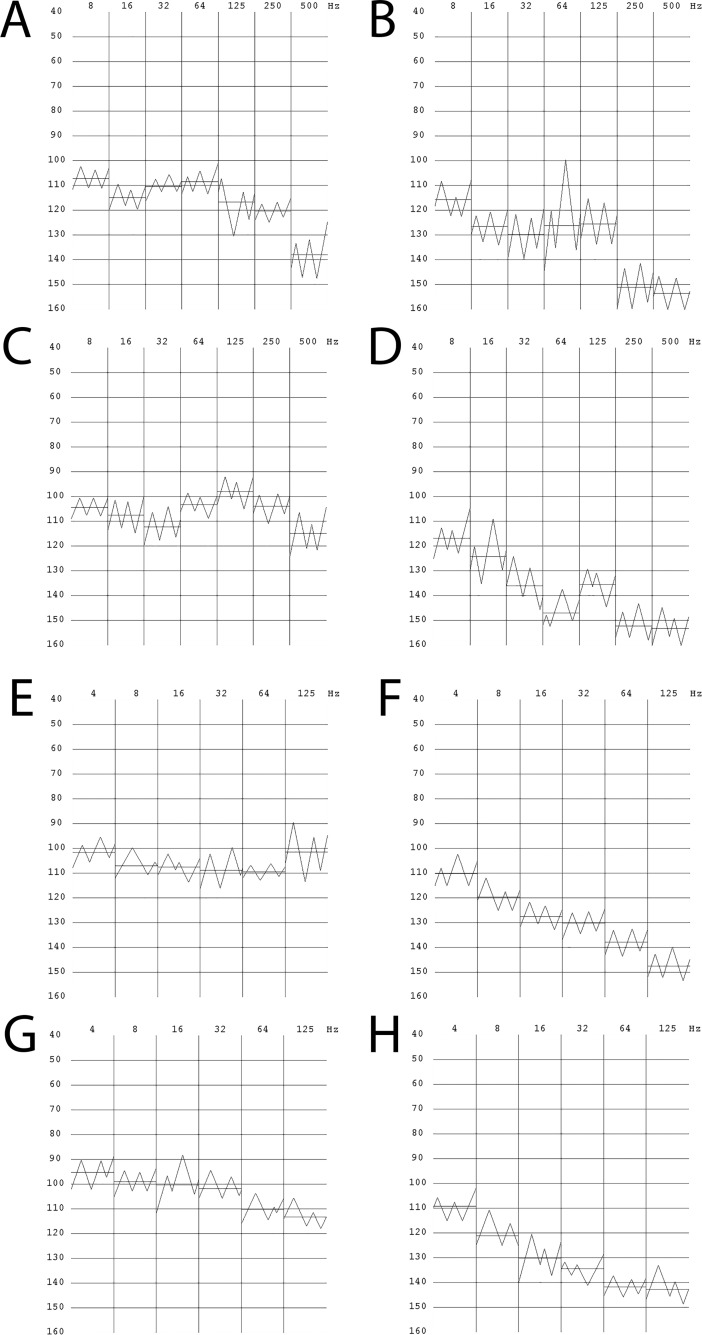
Vibrograms showing normal and abnormal VPTs. The vibrograms presented all arise from subjects with type 1 diabetes. (A) and (C) shows vibrograms reflecting normal vibrotactile sense, obtained from a 9-year old girl, at index and little fingers respectively. (B) and (D) shows vibrograms mirroring impaired vibrotactile sense, at index and little fingers respectively, in a 9-year old girl. (E) and (G) shows normal vibrograms, obtained from a 14-year old boy, at MTH 1 and MTH 5 respectively. (F) and (H) shows vibrograms, obtained from a 14-year old boy, mirroring impaired vibrotactile sense, at MTH 1 and MTH 5 respectively.

Z-scores for the numerical results of VPT assessments were calculated in comparison to previously collected normative data [21]. The resulting curves from the vibrometry examination were manually studied and visually abnormal frequencies were removed prior to statistical analyses. To be included for statistical analysis, each subject needed to present at least one visibly correct site, out of the four sites tested. Furthermore, each site being included for statistical analysis needed to have at least three visibly normal frequencies, i.e. the VPT curve needed to have at least five endpoints and the pattern needed to be structured, with no outlying endpoints reflecting lack of concentration. Examples of visibly incorrect vibrograms are shown in S1 Fig, and visibly correct vibrograms are shown in Fig 1. A site was considered pathological when at least three of the frequencies had z-scores of >1.96.

#### Perception to light touch

Using a 20 pieces Semmes-Weinstein’s monofilament collection [Touch-Test™, North Coast Medical Inc., Morgan Hill, Ca, USA; filaments ranging from 1.65 (0.008 g) to 6.65 (300 g)], LTP was assessed by applying the thinnest monofilament, size 1.65, with a firm, constant pressure to the same sites as VPTs were measured. If the subject was not able to perceive the stimulus the procedure was repeated with the next, thicker monofilament. This method was repeated until a positive response was achieved. In the hand, tactile sensitivity to ≤ 2.83 (0.07 g) were considered normal, and for the plantar surface of the foot normal tactile sensitivity was considered ≤ 3.61 (0.4 g), as recommended by the manufacturer, but lower than previously used thresholds in a study on pediatric subjects by Nelson et al. [23]

#### Statistical analyses

Data are presented as medians and quartiles for the entire study group, and for boys and girls, respectively. The study group was split into two groups, based on median disease duration [11]. Differences in characteristics and obtained VPT values between the groups were tested with non-parametric Mann-Whitney U-tests. When comparing VPT values, a Bonferroni correction was applied due to a large number of analyses (k = 24).

Correlations between subjects presenting with one or more sites considered pathological, due to previously stated criteria, and different characteristics of the subjects were performed using Chi2 test. All statistical analyses were made using IBM SPSS Statistics (Statistical Package for the Social Sciences, SPSS Inc., Chicago, Il, USA) version 23 for Mac.

## Results

### Subjects

Listed in Table 1 are the characteristics of the 72 children and adolescents (boys = 39) meeting the inclusion criteria. Median age was 12.8 [11.5–15.0] years and median disease duration was 5.3 [2.9–8.6] years. Median age at disease onset was 6.9 [4.7–10.3]. The median of last HbA1c values, prior to examination, among the subjects was 7.3 [6.7–7.8]% (57 [50–62] mmol/mol). A total of 45 subjects were treated with continuous subcutaneous insulin infusion (CSII) and 27 subjects were given insulin as multiple daily injections (MDI). Presented in Table 1 are also the characteristics for the two groups based on gender and median split of disease duration; i.e. less than, and more than 5.3 years.

**Table 1:** Characteristics of subjects.

Subjects Characteristics	All (n = 72) *	Boys (n = 39) †	Girls (n = 33) ‡	p-values §	Duration ≤ 5.3 years (n = 36) | |	Duration > 5.3 years (n = 36)	p-values ¶
**Age**	12.8 [11.5–15.0]	13.1 [10.9–14.8]	12.8 [11.7–15.2]	p = 0.848	12.0 [11.2–14.5]	14.0 [12.4–15.9]	**p = 0.011**
**Age at onset**	6.9 [4.7–10.3]	6.8 [4.8–10.8]	7.2 [4.3–10.1]	p = 0.861	9.3 [7.3–12.1]	4.9 [2.7–6.4]	**p < 0.001**
**Duration of disease**	5.3 [2.9–8.6]	5.4 [3.2–8.6]	5.3 [2.3–8.8]	p = 0.888	2.9 [1.3–3.9]	8.6 [7.0–10.2]	**p < 0.001**
**BMI SD**	0.51 [-0.17–1.29]	0.27 [-0.53–0.94]	1.00 [0.30–1.62]	**p = 0.005**	0.27 [-0.52–1.29]	0.75 [0.27–1.29]	p = 0.149
**HbA1c, last value** **% (mmol/mol)**	7.3 [6.7–7.8] (57 [50–62])	7.4 [6.7–7.9] (57 [50–63])	7.3 [6.8–7.6] (56 [51–60])	p = 0.991	7.0 [6.5–7.8] (54 [48–62])	7.4 [6.9–8.0] (57 [52–64])	p = 0.131
**HbA1c, 1-year mean** **% (mmol/mol)**	7.4 [6.9–7.8] (57 [52–62])	7.3 [6.9–7.7] 56 [51–61]	7.4 [7.0–7.9] (58 [53–63])	p = 0.462	7.2 [6.7–7.6] (56 [49–60])	7.5 [7.1–8.0] (58 [54–64])	**p = 0.023**
**HbA1c, 2-year mean** **% (mmol/mol)**	7.4 [6.9–7.8] (57 [52–62])	7.4 [6.9–7.7] 57.0 [52–61]	7.3 [7.1–7.9] (57 [54–63])	p = 0.614	7.1 [6.7–7.7] (54 [49–61])	7.5 [7.2–7.9] (58 [55–63])	**p = 0.019**
**Insulin administration:** **Insulin pump** **Insulin pen**	n = 45 n = 27	n = 26 n = 13	n = 19 n = 14		n = 18 n = 18	n = 27 n = 9	
**Insulin–IU/24h**	40.8 [25.7–56.4]	36.2 [23.8–51.3]	42.9 [29.4–63.6]	p = 0.229	34.8 [19.4–56.4]	46.1 [32.4–59.4]	p = 0.073
**Insulin–IU/kg/24h**	0.8 [0.6–1.0]	0.8 [0.6–1.0]	0.9 [0.6–1.0]	p = 0.523	0.8 [0.5–1.0]	0.9 [0.7–1.0]	p = 0.193

Values are expressed as medians [lower quartile–upper quartile]. HbA1c-values are given as % and due to IFCC standard in parenthesis (mmol/mol). Significant p-values at 0.05 level are in bold.

* n = 68 for “HbA1c, 1-year mean” and n = 63 for “HbA1c, 2-year mean”.

† n = 38 for “HbA1c, 1-year mean” and n = 35 for “HbA1c 2-year mean”.

‡ n = 30 for “HbA1c, 1-year mean” and n = 28 for “HbA1c, 2-year mean”.

§ Comparison of characteristics between boys and girls using Mann-Whitney U-test.

| | n = 32 for “HbA1c, 1-year mean” and n = 27 for “HbA1c, 2-year mean”.

¶ Comparison of characteristics between subjects with a disease duration of less than, and more than, 5.3 years using Mann-Whitney U-test.

### Vibration perception thresholds

Examples of vibrograms, obtained from the subjects with T1D, showing normal and impaired vibrotactile sense on all four sites examined are shown in Fig 1. All vibrograms presented graphically are age and gender matched, illustrating that children and adolescents with T1D can present with both normal and pathological vibrograms.

VPTs, presented as median z-scores, related to previously collected normative data (18), are shown in S1 (index finger), S2 (little finger) and S3 (MTH 1 and MTH 5 of the foot) Tables. Using Mann-Whitney U-tests with Bonferroni correction for multiple analyses (k = 24), on flagged significant values, showed no differences neither between boys and girls, nor between the subjects with a disease duration above or below median.

### Elevated VPTs in relation to LTPs

Due to the criteria stated above in the methods section, a total of 13 out of 72 subjects (18%) presented with at least one pathological site on the foot, and a total of two subjects (3%) showed general elevations of VPTs on all four sites examined. Three out of 72 subjects (4%) had three pathological sites, and four out of 72 subjects (6%) had two pathological sites. A total of 64 out of the 72 subjects were also examined with Semmes-Weinstein’s monofilaments. None of the subjects presented with impaired LTP at any of the sites examined.

### Elevated VPTs in the foot and hand

**Fig 2:**
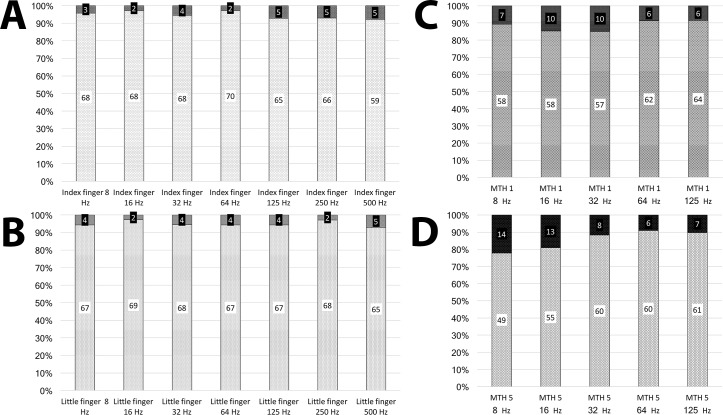
VPT graphs. These graphs show the number of subjects presenting with pathological (>1.96), and non-pathological (<1.96), z-scores at all examined frequencies and sites on the index **(A)** and little **(B)** fingers of the hand and on MTH 1 **(C)** and MTH 5 **(D)** on the foot. Z-scores are calculated based on normative values previously collected from healthy children and adolescents [21].

From a group perspective, our data determined a higher occurrence of pathological frequencies, i.e. z-score >1.96, from the VPT examinations than would be expected comparing the obtained VPTs to our normative data. With a confidence interval of 95%, only 2.5% of the subjects should have VPTs with z-scores of >1.96 unless impaired vibrotactile sense is present in the group. Among the studied sites in the hand, the proportion of pathological VPTs ranged from 2.8 to 7.8%. In the foot, the proportion varied from 8.6 to 22.2%, with the highest proportion of pathological values presenting at low frequencies on both MTH 1 and MTH 5 (Fig 2).

Out of the 13 subjects presenting with impaired vibrotactile sense, three subjects had concurrent impaired vibrotactile sense in the hand. Only one subject presented with impaired vibrotactile sense in the hand, without having impaired sense in the foot at the same time.

### Elevated VPTs in relation to treatment

**Fig 3:**
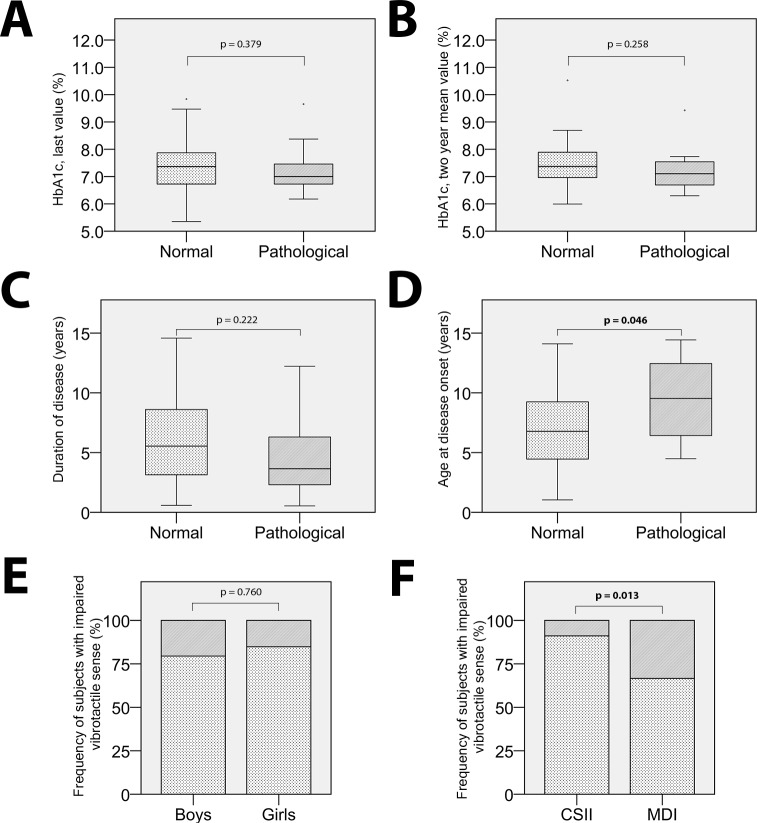
Boxplots and graphs. Neither last **(A)**, nor two-year mean **(B)**, HbA1c values differed between subjects with normal and impaired vibrotactile sense on at least one site on the foot. The duration of disease **(C)** did not statistically differ between subjects with normal and impaired vibrotactile sense on at least one site on the foot. However, among subjects with impaired vibrotactile sense, on at least one site of the foot, disease onset age was significantly higher **(D)**. The frequency of subjects with impaired vibrotactile sense, darker areas of the histograms, did not differ among boys and girls **(E)**, but subjects treated with MDI were more likely to have impaired vibrotactile sense **(F)**, than subjects treated with CSII. Among the 13 subjects with impaired vibrotactile sense four were receiving CSII treatment, and nine MDI treatment.

Pathological VPTs on at least one site on the foot, seen in 13 out of 72 subjects, were more common among subjects treated with MDI (n = 9) compared to patients with CSII (n = 4) treatment (p = 0.013) (Fig 3F). No differences were seen comparing boys and girls (p = 0.760) (Fig 3E).

General characteristics for the subjects treated with MDI and CSII is presented in S4 Table. Children treated with MDI had older age at onset (p = 0.024), shorter disease duration (p = 0.025), higher daily dose of insulin (p = 0.015), but similar HbA1c levels, compared to those treated with CSII.

### Elevated VPTs in relation to general characteristics

Median [quartiles] age, age at disease onset and disease duration of the subjects with at least one pathological site on the foot (n = 13) was 15.0 [11.5–16.8] years, 9.5 [5.7–12.6] years and 3.7 [1.8–7.1] years, respectively, and correspondingly among subjects with no pathological sites on the foot (n = 59) 12.7 [11.6–14.7] years, 6.8 [4.4–9.3] years and 5.5 [3.1–8.6] years, respectively. Age at disease onset was higher in the group of subjects presenting with at least one pathological site on the foot (p = 0.046) compared to the subjects with no pathological sites on the foot (Fig 3D). No such differences were seen comparing duration of disease (p = 0.222) (Fig 3C), last (p = 0.379) (Fig 3A) and two-year mean (p = 0.258) (Fig 3B) HbA1c, nor comparing age (p = 0.222), in groups with normal or abnormal VPTs. Furthermore, no differences were seen regarding normal or impaired vibrotactile sense in comparison with height (p = 0.263), weight (p = 0.353) or BMI SD (p = 0.356).

Screening for microalbuminuria in Sweden is regularly started at the age of 10. In 61 out of the 72 subjects, data on microalbuminuria was available. None of these 61 subjects, including all subjects with altered vibrotactile sense, showed signs of microalbuminuria at the time of vibrotactile examination. Blood pressure, available in 60 participants, were all within normal limits. Likewise, 67 out of 72 subjects, including all subjects with altered vibrotactile sense, had data on the presence of retinopathy in their medical records. Five subjects (7%) showed signs of mild retinopathy at the time of the vibrotactile examination, and two out of these five subjects showed concurrent impaired vibrotactile sense. Fishers exact test showed that retinopathy was not more common among subjects with neuropathy, and vice versa (p = 0.247).

## Discussion

In this study, we were, to our knowledge, for the first time investigating vibrotactile sense in children and adolescents with T1D, using multi-frequency vibrometry. A total of 13 out of 72 (18%) subjects presented with impaired vibrotactile sense on at least one site on the foot, compared to age and gender matched controls, showing us that it is possible to identify children and adolescents with signs of underlying DPN using multi-frequency vibrometry.

Our data clearly indicate that subjects treated with MDI were more likely to show signs of impaired vibrotactile sense than those treated with CSII, although they had similar quality of glycaemic control and shorter diabetes duration. This is similar to what was presented by Zabeen et al., showing that treatment with CSII is connected to lower rates of peripheral nerve abnormalities [24]. A possible explanation to this is that CSII is associated with a more even distribution of insulin than MDI that might not be reflected in HbA1c values. Another study has shown that CSII, compared to MDI, treatment was associated with regeneration of the corneal nerve fibers, although HbA1c values did not differ between the groups [25]. This is interesting and suggests that CSII treatment is somewhat connected to a better survival and growth environment for the nerve fibers. It is well established that increased metabolic control can decelerate the development of DPN in an adult population of subjects with T1D [26]. Furthermore, the most significant risk factor of developing DPN is poor metabolic control, reflected by increased HbA1c values [27]. In our study, we did not find any correlation between higher HbA1c values and the presence of elevated VPTs. A possible explanation might be that the subjects of our study are, in comparison to previous studies, presenting with lower HbA1c values. Additionally, since HbA1c reflects an average of the blood glucose level over time, short-timed spikes of high and low blood glucose levels may not be reflected. Our results support the theory that glycemic variability might play a role in DPN development in subjects with T1D [28–30]. However, in order to establish this correlation, it would be necessary to follow the subjects over time with at least two VPT examinations, with a substantially long follow up period, as well as equipping the subjects with continuous glucose monitors.

Furthermore, our data suggests that an older age at onset of T1D might somehow be connected to impaired vibrotactile sense on the foot. This might be explained by the better plasticity of peripheral nerves in younger children, compared to adolescents, as well as the brains ability to adapt to changes after nerve damage, as previously reported in children and adolescents undergoing nerve repair due to a median nerve injury [31, 32]. Therefore, screening for subclinical DPN in children and adolescents with T1D is important to enable an early detection and establishment of optimized glycemic control. Similarly, diabetic nephropathy, another well-known microangiopathic complication to T1D, has been shown to be more common among subjects with a later disease onset [33, 34].

Retinopathy is another well-known complication to T1D, and therefore children and adolescents are screened for this during child- and adulthood [15]. Previous studies have suggested that neuropathy, measured with corneal confocal microscopy, may precede both retinopathy and microalbuminuria in adults with T1D, and that small nerve fiber dysfunction may precede large nerve fiber dysfunction [35, 36]. As presented earlier, data on retinopathy was present in 67/72 subjects of this study. Only five subjects had retinopathy (all mild), and two out of these five subjects showed concurrent impaired vibrotactile sense, concluding that neuropathy, present in 13 out of 72 subjects, seem to be more common than retinopathy in our study.

In contrast to most studies, we chose to measure the VPTs and LTP on finger pulps and foot soles because these sites are tactile surfaces. Therefore, it is likely to believe that they better correlate to actions performed by the hands and feet than non-tactile surfaces. The presently used sites are different from the ones used in the reviewed articles by Hirschfeld et al., where VPTs were solely obtained from the feet, and not from hands [10]. In our study, we have shown that impaired vibrotactile sense is more common in the foot than in the hand, which supports the theory that the nerves in the lower limbs are affected by DPN before the nerves in the upper limbs, due to the length of the nerves [14]. Previous studies have also shown that nerve conduction amplitudes decrease with the height of the subject, but in our study we did not see any correlation between height and impaired vibrotactile sense [37].

A recent position statement for diabetic neuropathy, claims that a 128 Hz tuning fork can be used for assessment of vibration perception [13]. In Sweden, a 128 Hz tuning fork is recommended when screening for peripheral neuropathy among patients with diabetes, but this method has got a low sensitivity in the detection of DPN [13, 38, 39]. Since more pathological VPTs are present at 16 Hz than at 125 Hz, on both sites examined on the foot, questions are raised on the eligibility of using a 128 Hz tuning fork in regular screening for DPN.

The most obvious limitation to this study is that the subjects have not been examined with an electrophysiology method, measuring nerve conduction velocities and amplitudes. Although 13/72 subjects (18%) were identified with at least one pathological examined site on the foot, comparisons must be made between the VibroSense Meter and electrophysiology in order to establish the sensitivity of the method. However, the results of the measurements in the patients with T1D were compared to normal data from 269 school children aged eight to 20, and the finding should therefore be reliable. Another limitation is that the children and adolescents have not undergone a clinical neurological examination or examinations with regular screening tools, apart from monofilaments, such as a biothesiometer and tuning forks. Such examinations have been made by Blankenburg et al. (2012), and according to their findings tactile detection, using von Frey filaments, was a better screening tool for DPN than vibration testing, using a Rydel-Seifer tuning fork [11]. None of the children or adolescents in our study had any clinical signs of neuropathy, such as numbness or pain in palms or foot soles, according to their medical records. A possible weakness is that the subjects have only undergone one examination of their vibrotactile sense, but a previous study using the Vibrosense Meter has emphasized that there is a strong reliability of the technique in the test-retest of patients with neuropathy; in that case hand arm vibration syndrome (i.e. HAVS) [40].

The lack of data regarding Tanner stage of the subjects is a limitation that could possibly explain why subjects with older disease onset age, and thereby more likely to have entered puberty, present with a higher proportion of impaired vibrotactile sense. A similar correlation has been shown by Barkai et al., showing that puberty is a risk factor for diabetic neuropathy [41]. However, the subjects z-scores were compared with an age and gender matched healthy population of children and adolescents, where no Tanner stages were judged [21].

## Conclusions

Signs of DPN, not identified using Semmes-Weinstein’s monofilaments, can be detected in children and adolescents with T1D by the Vibrosense Meter. Since as many as 18% of the subjects had signs of DPN, screening is important. Further studies are needed to validate the finding of the increased risk of DPN with older age at onset and treatment with MDI compared to CSII.

## Supporting information

S1 Fig Example of visibly incorrect vibrograms. An example of a visibly incorrect frequency is shown in **(A)** at 500 Hz. Prior to statistical analysis the VPT value of 500 Hz is being excluded. **(B)** is showing a vibrogram being excluded in whole, due to the pattern of the curves, as well as the lack of VPT at 8 Hz. Even 4 and 250 Hz seem to be lacking in **(B)**, but these frequencies have not been examined used in the present examination.
(TIF)

S1 Table Z-scores of VPTs obtained from index finger. Median [lower quartile–upper quartile] values of z-scores from VPTs at all frequencies obtained from index finger on the right hand. Comparisons, using Mann Whitney U-tests, are made between boys and girls, and between subjects with a disease duration of less than and more than 5.3 years. P-values are presented and significant p-values, at 0.05 level, are corrected with Bonferroni corrections for multiple analyses (k = 24) and presented in parenthesis.
(DOCX)

S2 Table Z-scores of VPTs obtained from little finger. Median [lower quartile–upper quartile] values of z-scores from VPTs at all frequencies obtained from little finger on the right hand. Comparisons, using Mann Whitney U-tests, are made between boys and girls, and between subjects with a disease duration of less than and more than 5.3 years. P-values are presented and significant p-values, at 0.05 level, are corrected with Bonferroni corrections for multiple analyses (k = 24) and presented in parenthesis.
(DOCX)

S3 Table Z-scores of VPTs obtained from metatarsal heads one and five on the foot. Median [lower quartile–upper quartile] values of z-scores from VPTs at all frequencies obtained from MTH 1 and MTH 5 on the right foot. Comparisons, using Mann Whitney U-tests, are made between boys and girls, and between subjects with a disease duration of less than and more than 5.3 years. P-values are presented and significant p-values, at 0.05 level, are corrected with Bonferroni corrections for multiple analyses (k = 24) and presented in parenthesis.
(DOCX)

S4 Table Characteristics of subjects divided by treatment methods. Values are expressed as medians [lower quartile–upper quartile]. HbA1c-values are given as % and due to IFCC standard in parenthesis (mmol/mol). Significant p-values at 0.05 level are in bold.
* n = 42 for “HbA1c, 2-year mean”.
† n = 38 for “HbA1c, 1-year mean” and n = 35 for “HbA1c 2-year mean”.
‡ n = 23 for “HbA1c, 1-year mean” and n = 21 for “HbA1c, 2-year mean”.
§ Comparison of characteristics between subjects treated CSII and subjects treated with MDI using Mann-Whitney U-test.
(DOCX)

## Abbreviations

BMI SD: BMI standard deviation score
DPN: Diabetic peripheral neuropathy
LTP: Light-touch perception
MTH 1: First metatarsal head
MTH 5: Fifth metatarsal head
VPT: Vibration perception threshold

## References

[1] Tesfaye S, Boulton AJ, Dyck PJ, Freeman R, Horowitz M, Kempler P, et al Diabetic neuropathies: update on definitions, diagnostic criteria, estimation of severity, and treatments. Diabetes care. 2010;33(10):2285–93. Epub 2010/09/30. doi: 10.2337/dc10-1303 ; PubMed Central PMCID: PMCPMC2945176. 20876709 10.2337/dc10-1303 PMC2945176

[2] Bruce DG, Davis WA, Davis TM. Longitudinal predictors of reduced mobility and physical disability in patients with type 2 diabetes: the Fremantle Diabetes Study. Diabetes care. 2005;28(10):2441–7. Epub 2005/09/28. . 16186277 10.2337/diacare.28.10.2441

[3] Currie CJ, Poole CD, Woehl A, Morgan CL, Cawley S, Rousculp MD, et al The health-related utility and health-related quality of life of hospital-treated subjects with type 1 or type 2 diabetes with particular reference to differing severity of peripheral neuropathy. Diabetologia. 2006;49(10):2272–80. Epub 2006/09/01. doi: 10.1007/s00125-006-0380-7 . 16944094 10.1007/s00125-006-0380-7

[4] Kiziltan ME, Gunduz A, Kiziltan G, Akalin MA, Uzun N. Peripheral neuropathy in patients with diabetic foot ulcers: clinical and nerve conduction study. Journal of the neurological sciences. 2007;258(1–2):75–9. Epub 2007/04/03. doi: 10.1016/j.jns.2007.02.028 . 17399742 10.1016/j.jns.2007.02.028

[5] Morbach S, Lutale JK, Viswanathan V, Mollenberg J, Ochs HR, Rajashekar S, et al Regional differences in risk factors and clinical presentation of diabetic foot lesions. Diabetic medicine: a journal of the British Diabetic Association. 2004;21(1):91–5. Epub 2004/01/07. . 14706061 10.1046/j.1464-5491.2003.01069.x

[6] Schaper NC, Van Netten JJ, Apelqvist J, Lipsky BA, Bakker K. Prevention and management of foot problems in diabetes: A Summary Guidance for Daily Practice 2015, based on the IWGDF guidance documents. Diabetes research and clinical practice. 2017;124:84–92. Epub 2017/01/26. doi: 10.1016/j.diabres.2016.12.007 . 28119194 10.1016/j.diabres.2016.12.007

[7] Kerr M, Rayman G, Jeffcoate WJ. Cost of diabetic foot disease to the National Health Service in England. Diabetic medicine: a journal of the British Diabetic Association. 2014;31(12):1498–504. Epub 2014/07/06. doi: 10.1111/dme.12545 . 24984759 10.1111/dme.12545

[8] Weintrob N, Amitay I, Lilos P, Shalitin S, Lazar L, Josefsberg Z. Bedside neuropathy disability score compared to quantitative sensory testing for measurement of diabetic neuropathy in children, adolescents, and young adults with type 1 diabetes. Journal of diabetes and its complications. 2007;21(1):13–9. Epub 2006/12/27. doi: 10.1016/j.jdiacomp.2005.11.002 . 17189869 10.1016/j.jdiacomp.2005.11.002

[9] Louraki M, Karayianni C, Kanaka-Gantenbein C, Katsalouli M, Karavanaki K. Peripheral neuropathy in children with type 1 diabetes. Diabetes & metabolism. 2012;38(4):281–9. Epub 2012/04/17. doi: 10.1016/j.diabet.2012.02.006 . 22503144 10.1016/j.diabet.2012.02.006

[10] Hirschfeld G, von Glischinski M, Blankenburg M, Zernikow B. Screening for peripheral neuropathies in children with diabetes: a systematic review. Pediatrics. 2014;133(5):e1324–30. Epub 2014/04/09. doi: 10.1542/peds.2013-3645 . 24709928 10.1542/peds.2013-3645

[11] Blankenburg M, Kraemer N, Hirschfeld G, Krumova EK, Maier C, Hechler T, et al Childhood diabetic neuropathy: functional impairment and non-invasive screening assessment. Diabetic medicine: a journal of the British Diabetic Association. 2012;29(11):1425–32. Epub 2012/04/18. doi: 10.1111/j.1464-5491.2012.03685.x . 22507184 10.1111/j.1464-5491.2012.03685.x

[12] Hirschfeld G, von Glischinski M, Knop C, Wiesel T, Reinehr T, Aksu F, et al Difficulties in screening for peripheral neuropathies in children with diabetes. Diabetic medicine: a journal of the British Diabetic Association. 2015;32(6):786–9. Epub 2015/02/03. doi: 10.1111/dme.12684 . 25640325 10.1111/dme.12684

[13] Pop-Busui R, Boulton AJ, Feldman EL, Bril V, Freeman R, Malik RA, et al Diabetic Neuropathy: A Position Statement by the American Diabetes Association. Diabetes care. 2017;40(1):136–54. Epub 2016/12/22. doi: 10.2337/dc16-2042 . 27999003 10.2337/dc16-2042 PMC6977405

[14] Louraki M, Tsentidis C, Kallinikou D, Katsalouli M, Kanaka-Gantenbein C, Kafassi N, et al Reproducibility of vibration perception threshold values in children and adolescents with type 1 diabetes mellitus and associated factors. Primary care diabetes. 2014;8(2):147–57. Epub 2013/12/10. doi: 10.1016/j.pcd.2013.11.002 . 24315733 10.1016/j.pcd.2013.11.002

[15] Donaghue KC, Wadwa RP, Dimeglio LA, Wong TY, Chiarelli F, Marcovecchio ML, et al ISPAD Clinical Practice Consensus Guidelines 2014. Microvascular and macrovascular complications in children and adolescents. Pediatric diabetes. 2014;15 Suppl 20:257–69. Epub 2014/09/04. doi: 10.1111/pedi.12180 . 25182318 10.1111/pedi.12180

[16] Consensus statement: Report and recommendations of the San Antonio conference on diabetic neuropathy. American Diabetes Association American Academy of Neurology. Diabetes care. 1988;11(7):592–7. Epub 1988/07/01. . 3060328 10.2337/diacare.11.7.592

[17] Johansson RS, Vallbo ÅB. Tactile sensory coding in the glabrous skin of the human hand. Trends in Neurosciences. 1983;6:27–32. http://dx.doi.org/10.1016/0166-2236(83)90011-5.

[18] Bell J, Bolanowski S, Holmes MH. The structure and function of Pacinian corpuscles: a review. Progress in neurobiology. 1994;42(1):79–128. Epub 1994/01/01. . 7480788 10.1016/0301-0082(94)90022-1

[19] Sato M. Response of Pacinian corpuscles to sinusoidal vibration. The Journal of physiology. 1961;159:391–409. Epub 1961/12/01. ; PubMed Central PMCID: PMCPmc1359541. 14497425 10.1113/jphysiol.1961.sp006817 PMC1359541

[20] Dahlin E, Ekholm E, Gottsater A, Speidel T, Dahlin LB. Impaired vibrotactile sense at low frequencies in fingers in autoantibody positive and negative diabetes. Diabetes research and clinical practice. 2013;100(2):e46–50. Epub 2013/03/08. doi: 10.1016/j.diabres.2013.01.026 . 23465366 10.1016/j.diabres.2013.01.026

[21] Dahlin LB, Guner N, Elding Larsson H, Speidel T. Vibrotactile perception in finger pulps and in the sole of the foot in healthy subjects among children or adolescents. PloS one. 2015;10(3):e0119753 Epub 2015/04/04. doi: 10.1371/journal.pone.0119753 ; PubMed Central PMCID: PMCPMC4383580. 25835710 10.1371/journal.pone.0119753 PMC4383580

[22] Nelander J, Speidel T, Bjorkman A, Dahlin LB. Vibration thresholds are increased at low frequencies in the sole of the foot in diabetes-a novel multi-frequency approach. Diabetic medicine: a journal of the British Diabetic Association. 2012;29(12):e449–56. Epub 2012/09/25. doi: 10.1111/dme.12024 . 22998552 10.1111/dme.12024

[23] Nelson D, Mah JK, Adams C, Hui S, Crawford S, Darwish H, et al Comparison of conventional and non-invasive techniques for the early identification of diabetic neuropathy in children and adolescents with type 1 diabetes. Pediatric diabetes. 2006;7(6):305–10. Epub 2007/01/11. doi: 10.1111/j.1399-5448.2006.00208.x . 17212597 10.1111/j.1399-5448.2006.00208.x

[24] Zabeen B, Craig ME, Virk SA, Pryke A, Chan AKF, Cho YH, et al Insulin pump therapy is associated with lower rates of retinopathy and peripheral nerve abnormality. PloS one. 2016;11(4). doi: 10.1371/journal.pone.0153033 27050468 10.1371/journal.pone.0153033 PMC4822832

[25] Azmi S, Ferdousi M, Petropoulos IN, Ponirakis G, Fadavi H, Tavakoli M, et al Corneal confocal microscopy shows an improvement in small-fiber neuropathy in subjects with type 1 diabetes on continuous subcutaneous insulin infusion compared with multiple daily injection. Diabetes care. 2015;38(1):e3–4. Epub 2014/12/30. doi: 10.2337/dc14-1698 . 25538321 10.2337/dc14-1698

[26] Callaghan BC, Little AA, Feldman EL, Hughes RA. Enhanced glucose control for preventing and treating diabetic neuropathy. The Cochrane database of systematic reviews. 2012;(6):Cd007543 Epub 2012/06/15. doi: 10.1002/14651858.CD007543.pub2 ; PubMed Central PMCID: PMCPMC4048127. 22696371 10.1002/14651858.CD007543.pub2 PMC4048127

[27] Hajas G, Kissova V, Tirpakova A. A 10-yr follow-up study for the detection of peripheral neuropathy in young patients with type 1 diabetes. Pediatric diabetes. 2016;17(8):632–41. Epub 2016/11/04. doi: 10.1111/pedi.12382 . 27028140 10.1111/pedi.12382

[28] Siegelaar SE, Kilpatrick ES, Rigby AS, Atkin SL, Hoekstra JB, Devries JH. Glucose variability does not contribute to the development of peripheral and autonomic neuropathy in type 1 diabetes: data from the DCCT. Diabetologia. 2009;52(10):2229–32. Epub 2009/08/13. doi: 10.1007/s00125-009-1473-x ; PubMed Central PMCID: PMCPMC2744825. 19672575 10.1007/s00125-009-1473-x PMC2744825

[29] Soupal J, Skrha J Jr., Fajmon M, Horova E, Mraz M, Skrha J, et al Glycemic variability is higher in type 1 diabetes patients with microvascular complications irrespective of glycemic control. Diabetes technology & therapeutics. 2014;16(4):198–203. Epub 2014/01/10. doi: 10.1089/dia.2013.0205 . 24401008 10.1089/dia.2013.0205

[30] Bragd J, Adamson U, Backlund LB, Lins PE, Moberg E, Oskarsson P. Can glycaemic variability, as calculated from blood glucose self-monitoring, predict the development of complications in type 1 diabetes over a decade? Diabetes & metabolism. 2008;34(6 Pt 1):612–6. Epub 2008/10/01. doi: 10.1016/j.diabet.2008.04.005 . 18824382 10.1016/j.diabet.2008.04.005

[31] Chemnitz A, Bjorkman A, Dahlin LB, Rosen B. Functional outcome thirty years after median and ulnar nerve repair in childhood and adolescence. The Journal of bone and joint surgery American volume. 2013;95(4):329–37. Epub 2013/02/22. doi: 10.2106/JBJS.L.00074 . 23426767 10.2106/JBJS.L.00074

[32] Chemnitz A, Weibull A, Rosen B, Andersson G, Dahlin LB, Bjorkman A. Normalized activation in the somatosensory cortex 30 years following nerve repair in children: an fMRI study. The European journal of neuroscience. 2015;42(4):2022–7. Epub 2015/04/14. doi: 10.1111/ejn.12917 . 25865600 10.1111/ejn.12917

[33] Mollsten A, Svensson M, Waernbaum I, Berhan Y, Schon S, Nystrom L, et al Cumulative risk, age at onset, and sex-specific differences for developing end-stage renal disease in young patients with type 1 diabetes: a nationwide population-based cohort study. Diabetes. 2010;59(7):1803–8. Epub 2010/04/29. doi: 10.2337/db09-1744 ; PubMed Central PMCID: PMCPMC2889782. 20424230 10.2337/db09-1744 PMC2889782

[34] Finne P, Reunanen A, Stenman S, Groop PH, Gronhagen-Riska C. Incidence of end-stage renal disease in patients with type 1 diabetes. Jama. 2005;294(14):1782–7. Epub 2005/10/13. doi: 10.1001/jama.294.14.1782 . 16219881 10.1001/jama.294.14.1782

[35] Petropoulos IN, Green P, Chan AW, Alam U, Fadavi H, Marshall A, et al Corneal confocal microscopy detects neuropathy in patients with type 1 diabetes without retinopathy or microalbuminuria. PloS one. 2015;10(4):e0123517 Epub 2015/04/09. doi: 10.1371/journal.pone.0123517 ; PubMed Central PMCID: PMCPMC4390357. 25853247 10.1371/journal.pone.0123517 PMC4390357

[36] Szalai E, Deak E, Modis L Jr., Nemeth G, Berta A, Nagy A, et al Early Corneal Cellular and Nerve Fiber Pathology in Young Patients With Type 1 Diabetes Mellitus Identified Using Corneal Confocal Microscopy. Investigative ophthalmology & visual science. 2016;57(3):853–8. Epub 2016/03/05. doi: 10.1167/iovs.15-18735 . 26943147 10.1167/iovs.15-18735

[37] Cinar N, Sahin S, Sahin M, Okluoglu T, Karsidag S. Effects of anthropometric factors on nerve conduction: an electrophysiologic study of feet. Journal of the American Podiatric Medical Association. 2013;103(1):43–9. Epub 2013/01/19. . 23328852 10.7547/1030043

[38] The National Board of Health and Welfare S. National Guidelines for Diabetes Care—Support for governance and management 2015. Available from: http://www.socialstyrelsen.se/Lists/Artikelkatalog/Attachments/19803/2015-4-12.pdf.

[39] The National Board of Health and Welfare S. Nationella riktlinjer för diabetesvård–Stöd för styrning och ledning The National Board of Health and Welfare, Sweden; 2017. 116]. Available from: http://www.socialstyrelsen.se/SiteCollectionDocuments/2017-5-31-nr-diabetes-vetenskapligt-underlag.pdf.

[40] Gerhardsson L, Gillstrom L, Hagberg M. Test-retest reliability of neurophysiological tests of hand-arm vibration syndrome in vibration exposed workers and unexposed referents. Journal of occupational medicine and toxicology (London, England). 2014;9(1):38 Epub 2014/11/18. doi: 10.1186/s12995-014-0038-1 ; PubMed Central PMCID: PMCPMC4232643. 25400687 10.1186/s12995-014-0038-1 PMC4232643

[41] Barkai L, Kempler P. Puberty as a risk factor for diabetic neuropathy. Diabetes care. 2000;23(7):1044–5. Epub 2000/07/15. . 10895880 10.2337/diacare.23.7.1044

