# Supplementary material for: Impaired vibrotactile sense in children and adolescents with type 1 diabetes – Signs of peripheral neuropathy
Source: PLoS One. 2018 Apr 19;13(4):e0196243. doi: 10.1371/journal.pone.0196243 (PMC5908163; doi:10.1371/journal.pone.0196243)
Supplement: S1 Table — Median [lower quartile–upper quartile] values of z-scores from VPTs at all frequencies obtained from index finger on the right hand. Comparisons, using Mann Whitney U-tests, are made between boys and girls, and between subjects with a disease duration of less than and more than 5.3 years. P-values are presented and significant p-values, at 0.05 level, are corrected with Bonferroni corrections for multiple analyses (k = 24) and presented in parenthesis. (DOCX) [file pone.0196243.s002.docx]

***Supplemental Table S1.*** *Z-scores of VPTs obtained from index finger.*

| **Subjects**  **Site and**  **frequency** | | **All**  **(n=72)** | **Boys**  **(n=39)** | **Girls**  **(n=33)** | **p-values (Bonferroni corrected)** | **Duration**  **< 5.3 years**  **(n=36)** | **Duration**  **> 5.3 years**  **(n=36)** | **p-values (Bonferroni corrected)** |
| --- | --- | --- | --- | --- | --- | --- | --- | --- |
| **Index finger** | **8 Hz** | -0.160 [-0.710 – 0.680] | -0.200  [-0.830 – 0.760] | -0.130  [-0.625 – 0.613] | p = 0.844 | -0.245  [-0.740 – 0.520] | 0.020  [-0.660 – 0.760] | p = 0.414 |
| **Index finger** | **16 Hz** | -0.125 [-0.860 – 0.745] | -0.250  [-1.335 – 0.733] | 0.125  [-0.445 – 0.755] | p = 0.236 | -0.120  [-0.550 – 0.720] | -0.130  [-1.140 – 0.770] | p = 0.518 |
| **Index finger** | **32 Hz** | -0.695 [-1.520 – 0.363] | -0.560  [-1.960 – 0.390] | -0.710  [-1.375 – 0.385] | p = 0.778 | -0.480  [-1.180 – 0.543] | -1.075  [-2.060 – 0.165] | p = 0.037 (0.888) |
| **Index finger** | **64 Hz** | -0.725 [-1.628 – 0.158] | -1.210  [-1.750 – 0.250] | -0.610  [-1.440 – -0.070] | p = 0.480 | -0.570  [-1.530 – 0.603] | -1.085  [-1.918 – -0.108] | p = 0.091 |
| **Index finger** | **125 Hz** | -0.390 [-1.185 – 0.465] | -0.830  [-1.360 – 0.330] | -0.100  [-0.705 – 0.490] | p = 0.027 (0.648) | -0.180  [-1.155 – 0.668] | -0.675  [-1.305 – 0.330] | p = 0.175 |
| **Index finger** | **250 Hz** | -0.090  [-0.8890 – 0.330] | -0.595  [-1.293 – 0.298] | 0.080  [-0.445 – 0.600] | p = 0.035 (0.840) | -0.120  [-1.060 – 0.270] | -0.070  [-0.763 – 0.615] | p = 0.581 |
| **Index finger** | **500 Hz** | -0.115  [-0.820 – 0.470] | -0.110  [-0.790 – 0.380] | -0.250  [-0.840 – 0.700] | p = 0.968 | -0.080  [-0.510 – 0.570] | -0.290  [-0.860 – 0.560] | p = 0.292 |

***Supplemental Table S1.*** *Z-scores of VPTs obtained from index finger.*

Median [lower quartile – upper quartile] values of z-scores from VPTs at all frequencies obtained from index finger on the right hand. Comparisons, using Mann Whitney U-tests, are made between boys and girls, and between subjects with a disease duration of less than and more than 5.3 years. P-values are presented and significant p-values, at 0.05 level, are corrected with Bonferroni corrections for multiple analyses (k=24) and presented in parenthesis.
