# Supplementary material for: Impaired vibrotactile sense in children and adolescents with type 1 diabetes – Signs of peripheral neuropathy
Source: PLoS One. 2018 Apr 19;13(4):e0196243. doi: 10.1371/journal.pone.0196243 (PMC5908163; doi:10.1371/journal.pone.0196243)
Supplement: S2 Table — Median [lower quartile–upper quartile] values of z-scores from VPTs at all frequencies obtained from little finger on the right hand. Comparisons, using Mann Whitney U-tests, are made between boys and girls, and between subjects with a disease duration of less than and more than 5.3 years. P-values are presented and significant p-values, at 0.05 level, are corrected with Bonferroni corrections for multiple analyses (k = 24) and presented in parenthesis. (DOCX) [file pone.0196243.s003.docx]

***Supplemental Table S2.*** *Z-scores of VPTs obtained from little finger.*

| **Subjects**    **Site and**  **frequency** | | **All**  **(n=72)** | **Boys**  **(n=39)** | **Girls**  **(n=33)** | **p-values (Bonferroni corrected)** | **Duration**  **< 5.3 years**  **(n=36)** | **Duration**  **> 5.3 years**  **(n=36)** | **p-values (Bonferroni corrected)** |
| --- | --- | --- | --- | --- | --- | --- | --- | --- |
| **Little finger** | **8 Hz** | -0.080  [-0.740 – 0.640] | -0.380  [-1.160 – 0.528] | 0.060  [-0.545 – 1.155] | p = 0.091 | -0.160  [-0.560 – 0.500] | 0.060  [-1.025– 0.923] | p = 0.890 |
| **Little finger** | **16 Hz** | -0.370  [-1.070– 0.220] | -0.405  [-0.988 – 0.165] | -0.230  [-1.355 – 0.265] | p = 0.940 | -0.370  [-0.790 – 0.220] | -0.380  [-1.380 – 0.230] | p = 0.721 |
| **Little finger** | **32 Hz** | -0.430  [-1.305 – 0.548] | -0.430  [-1.350 – 0.410] | -0.570  [-1.245 – 0.675] | p = 0.591 | 0.075  [-0.958 – 0.725] | -0.830  [-1.838 – 0.033] | p = 0.013 (0.312) |
| **Little finger** | **64 Hz** | -0.630  [-1.280 – 0.090] | -0.815  [-1.560 – 0.165] | -0.540  [-1.225 – 0.110] | p = 0.464 | -0.625  [-1.213 – 0.400] | -0.680  [-1.460 – -0.120] | p = 0.455 |
| **Little finger** | **125 Hz** | -0.570  [-0.990 – 0.170] | -0.680  [-1.323 – 0.155] | -0.320  [-0.725 – 0.325] | p = 0.087 | -0.350  [-0.990 – 0.010] | -0.635  [-0.975 – 0.260] | p = 0.633 |
| **Little finger** | **250 Hz** | -0.435  [-0.940 – 0.225] | -0.700  [-1.180 – 0.195] | -0.260  [-0.905 – 0.370] | p = 0.067 | -0.480  [-1.030 – -0.090] | -0.315  [-0.895 – 0.338] | p = 0.269 |
| **Little finger** | **500 Hz** | -0.510  [-0.925 – 0.350] | -0.650  [-1.120 – 0.130] | -0.410  [-0.860 – 0.525] | p = 0.116 | -0.450  [-0.940 – 0.680] | -0.530  [-0.920 – 0.340] | p = 0.660 |

***Supplemental Table S2.*** *Z-scores of VPTs obtained from little finger.*

Median [lower quartile – upper quartile] values of z-scores from VPTs at all frequencies obtained from little finger on the right hand. Comparisons, using Mann Whitney U-tests, are made between boys and girls, and between subjects with a disease duration of less than and more than 5.3 years. P-values are presented and significant p-values, at 0.05 level, are corrected with Bonferroni corrections for multiple analyses (k=24) and presented in parenthesis.
