# Supplementary material for: Impaired vibrotactile sense in children and adolescents with type 1 diabetes – Signs of peripheral neuropathy
Source: PLoS One. 2018 Apr 19;13(4):e0196243. doi: 10.1371/journal.pone.0196243 (PMC5908163; doi:10.1371/journal.pone.0196243)
Supplement: S3 Table — Median [lower quartile–upper quartile] values of z-scores from VPTs at all frequencies obtained from MTH 1 and MTH 5 on the right foot. Comparisons, using Mann Whitney U-tests, are made between boys and girls, and between subjects with a disease duration of less than and more than 5.3 years. P-values are presented and significant p-values, at 0.05 level, are corrected with Bonferroni corrections for multiple analyses (k = 24) and presented in parenthesis. (DOCX) [file pone.0196243.s004.docx]

***Supplemental Table S3.*** *Z-scores of VPTs obtained from metatarsal heads one and five on the foot.*

| **Subjects**  **Site and**  **frequency** | | **All**  **(n=72)** | **Boys**  **(n=39)** | **Girls**  **(n=33)** | **p-values**  **(Bonferroni corrected)** | **Duration**  **< 5.3 years**  **(n=36)** | **Duration**  **> 5.3 years**  **(n=36)** | **p-values**  **(Bonferroni corrected)** |
| --- | --- | --- | --- | --- | --- | --- | --- | --- |
| **MTH 1** | **8 Hz** | -0.070  [-0.625 – 0.835] | -0.170  [-0.170 – 1.210] | -0.200  [-0.210 – 0.768] | p = 0.390 | -0.070  [-0.363 – 1.630] | -0.050  [-0.885– 0.700] | p = 0.355 |
| **MTH 1** | **16 Hz** | 0.370  [-0.520 – 1.065] | 0.370  [-0.878 – 1.633] | 0.365  [0.110 – 1.000] | p = 0.559 | 0.460  [-0.290 – 1.850] | 0.330  [-0.675 – 0.805] | p = 0.185 |
| **MTH 1** | **32 Hz** | 0.160  [-0.540 – 1.350] | -0.080  [-0.580 – 1.280] | 0.580  [-0.418 – 1.468] | p = 0.203 | 0.465  [-0.423 – 1.973] | -0.080  [-0.655 – 0.855] | p = 0.078 |
| **MTH 1** | **64 Hz** | 0.050  [-0.768 – 1.100] | -0.400  [-1.140 – 1.430] | 0.260  [-0.440 – 0.860] | p = 0.362 | 0.340  [-0.590 – 1.430] | -0.410  [-1.060 – 0.985] | p = 0.103 |
| **MTH 1** | **125 Hz** | -0.290  [-1.005 – 0.615] | -0.510  [-1.195 – 0.965] | 0.220  [-0.695 – 0.573] | p = 0.172 | -0.030  [-1.058 – 0.558] | -0.340  [-0.980 – 0.760] | p = 0.714 |
| **MTH 5** | **8 Hz** | 0.570  [-0.580 – 1.900] | 0.610  [-0.990 – 2.495] | 0.385  [-0.540 – 1.528] | p = 0.573 | 0.950  [-0.673 – 2.153] | 0.070  [-0.750 – 0.870] | p = 0.074 |
| **MTH 5** | **16 Hz** | 0.560  [-0.590 – 1.613] | 0.560  [-0.598 – 2.430] | 0.515  [-0.528 – 1.448] | p = 0.602 | 0.920  [-0.595– 1.775] | 0.240  [-0.590 – 1.310] | p = 0.138 |
| **MTH 5** | **32 Hz** | 0.200  [-0.648 – 0.943] | -0.150  [-0.910 – 0.970] | 0.300  [-0.465 – 0.925] | p = 0.637 | 0.250  [-0.603 – 1.193] | -0.120  [-0.920 – 0.790] | p = 0.267 |
| **MTH 5** | **64 Hz** | -0.080  [-0.715 – 0.888] | -0.370  [-0.860 – 0.900] | 0.080  [-0.435– 0.980] | p = 0.330 | 0.145  [-0.508– 1.070] | -0.320  [-0.868 – 0.880] | p = 0.149 |
| **MTH 5** | **125 Hz** | -0.115  [-0.753 – 0.640] | -0.290  [-0.870 – 0.750] | 0.175  [-0.645 – 0.640] | p = 0.435 | -0.190  [-0.790 – 0.570] | -0.040  [-0.660 – 0.990] | p = 0.560 |

***Supplemental Table S3.*** *Z-scores of VPTs obtained from metatarsal heads one and five on the foot.*

Median [lower quartile – upper quartile] values of z-scores from VPTs at all frequencies obtained from MTH 1 and MTH 5 on the right foot. Comparisons, using Mann Whitney U-tests, are made between boys and girls, and between subjects with a disease duration of less than and more than 5.3 years. P-values are presented and significant p-values, at 0.05 level, are corrected with Bonferroni corrections for multiple analyses (k=24) and presented in parenthesis.
