# Supplementary material for: Impaired vibrotactile sense in children and adolescents with type 1 diabetes – Signs of peripheral neuropathy
Source: PLoS One. 2018 Apr 19;13(4):e0196243. doi: 10.1371/journal.pone.0196243 (PMC5908163; doi:10.1371/journal.pone.0196243)
Supplement: S4 Table — Values are expressed as medians [lower quartile–upper quartile]. HbA1c-values are given as % and due to IFCC standard in parenthesis (mmol/mol). Significant p-values at 0.05 level are in bold. * n = 42 for “HbA1c, 2-year mean”. † n = 38 for “HbA1c, 1-year mean” and n = 35 for “HbA1c 2-year mean”. ‡ n = 23 for “HbA1c, 1-year mean” and n = 21 for “HbA1c, 2-year mean”. § Comparison of characteristics between subjects treated CSII and subjects treated with MDI using Mann-Whitney U-test. (DOCX) [file pone.0196243.s005.docx]

***Supplemental table S4.*** *Characteristics of subjects divided by treatment methods.*

| **Subjects**  **Characteristics** | **All (n = 72) *** | **CSII (n = 45) †** | **MDI (n = 27) ‡** | **p-values §** |
| --- | --- | --- | --- | --- |
| **Age** | 12.8 [11.5–15.0] | 12.6 [11.7–14.5] | 13.5 [11.5–15.8] | p=0.471 |
| **Age at onset** | 6.9 [4.7–10.3] | 6.4 [4.6–8.4] | 9.2 [4.7–12.5] | **p=0.024** |
| **Duration of disease** | 5.3 [2.9–8.6] | 5.6 [3.7–8.6] | 3.3 [1.2–7.9] | **p=0.025** |
| **BMI SD** | 0.51 [-0.17–1.29] | 0.46 [-0.23–1.29] | 0.68 [-0.14–1.29] | p=0.629 |
| **HbA1c, last value**  **% (mmol/mol)** | 7.3 [6.7–7.8]  (57 [50–62]) | 7.4 [6.7–7.9]  (57 [50–63]) | 7.1 [6.7–7.6]  (54 [50–60]) | p=0.399 |
| **HbA1c, 1-year mean**  **% (mmol/mol)** | 7.4 [6.9–7.8]  (57 [52–62]) | 7.3 [6.9–8.0]  (57 [52–63]) | 7.5 [6.9–7.7]  (58 [52–61]) | p=0.751 |
| **HbA1c, 2-year mean**  **% (mmol/mol)** | 7.4 [6.9–7.8]  (57 [52–62]) | 7.4 [6.9–7.9]  (57 [52–63]) | 7.3 [6.9–7.7]  (56 [52–61]) | p=0.726 |
| **Insulin administration:**  **Insulin pump**  **Insulin pen** | n = 45  n = 27 |  |  |  |
| **Insulin – IU/24h** | 40.8 [25.7–56.4] | 38.9 [24.4–51.3] | 53.0 [28.0–82.0] | p=0.095 |
| **Insulin – IU/kg/24h** | 0.8 [0.6–1.0] | 0.8 [0.6–0.9] | 1.0 [0.6–1.3] | **p=0.015** |

***Supplemental table S4.*** *Characteristics of subjects divided by treatment methods.*

Values are expressed as medians [lower quartile – upper quartile]. HbA1c-values are given as % and due to IFCC standard in parenthesis (mmol/mol). Significant p-values at 0.05 level are in bold.

* n = 42 for “HbA1c, 2-year mean”.

† n = 38 for “HbA1c, 1-year mean” and n = 35 for “HbA1c 2-year mean”.

‡ n = 23 for “HbA1c, 1-year mean” and n = 21 for “HbA1c, 2-year mean”.

§ Comparison of characteristics between subjects treated CSII and subjects treated with MDI using Mann-Whitney U-test.
